# A hybrid GNA instability

**DOI:** 10.1038/s41598-022-23854-x

**Published:** 2022-11-21

**Authors:** Pralay Kumar Karmakar, Dhrubajit Kalita, Ahmed Atteya

**Affiliations:** 1 Department of Physics, Tezpur University, Napaam, Tezpur, Assam 784028 India; 2 Department of Physics, North Gauhati College, College Nagar, Kamrup (R), Guwahati, Assam 781031 India; 3 Department of Physics, Faculty of Science, Alexandria University, P.O. 21511, Alexandria, Egypt

**Keywords:** Astronomy and planetary science, Physics

## Abstract

A semi-analytic admixed model formalism to study the stability effects of the inner crust regions against the local collective perturbations in non-rotating neutron stars is proposed. It consists of the viscoelastic heavy neutron-rich nuclei, superfluid neutrons, and degenerate quantum electrons. A normal spherical mode analysis yields a generalized linear dispersion relation multiparametrically mimicking the inner crust features of neutron stars. A hybrid gravito-nucleo-acoustic (GNA) instability mode is found to be excited. It is demonstrated that the electron density and the inner crust curvature act as its accelerating and antidispersive agents. In contrast, the heavy neutron-rich nucleus and neutron densities act as decelerating factors. The heavy nucleus density, electron density, and geometric curvature act as its destabilizers. It is only the neutron density that acts as the GNA stabilizing agent. The heavy neutron-rich nucleus and neutron densities are found to act as dispersive broadening factors to it. The high-$K$ regions are the more unstable spectral windows indicating that the GNA mode plays a dominant role in the inner crust zone towards the local stability. Its fair reliability is indicated in light of the recent astronomic observed scenarios. It could be useful to explore acoustic mode signatures in non-rotating neutron stars and similar other compact astroobjects.

## Introduction

A neutron star is a seismically active compact astrophysical remnant object composed mainly of degenerate nuclear matter in spherically confined geometry^1,2^. It is formed from the gravitational core-collapse of massive stars ($M\sim (8 - 10)\,M_{\Theta }$, $M_{\Theta } = 2 \times 10^{30}$ kg is the solar mass). Its organizing energy of the gravitational pull is balanced by the disorganizing elastic energy stored in the neutron Fermi-continuum^3^. The typical physical properties of such astroobjects are mass^4,5^, $M\sim (1.4 - 2.14)\,M_{\Theta }$; size, $R\sim 10 - 20$ km^1,2^, temperature^2,6^, $T\sim 10^{6} - 10^{12}$ K, and so forth. The internal structure of such stars are revealed by analysing the observed spectra of neutron star oscillation. Its interior structure is categorically subdivided into five distinct concentric regions on the basis of its compositional matter density $(\rho )$^2,7^. These constituent regions are: (1) thin atmosphere of light elements surrounding an ocean of superhot liquid iron; (2) an outer crust composed of dense plasma of neutron-rich nuclei and quantum degenerate electron gas ($\rho \sim 10^{7} - 10^{14}$ kg m^−3^); (3) inner crust composed of inhomogeneous neutron-rich nuclei, neutron superfluid, and electron quantum fluid ($\rho \sim 10^{14} - 10^{17}$ kg m^−3^); (4) outer core made of neutrons, non-degenerate protons and muons; and (5) abstract inner core^2,7^.

The dynamics of the interior of the neutron star is important to understand a rich variety of phenomena, such as observed spin glitches, thermal evolution, waves and oscillations, and diversified instabilities^8^. In this regard, the importance of hydrodynamic instabilities in such neutron stars has become a question of considerable interest. It may be surprisingly noted that the inner crust region has received only a little research attention as of now from the fluidic stability viewpoint. In the inner crust, if $\rho = 4.3 \times 10^{14}$ kg m^−3^, neutrons drip out of the neutron-rich nuclei and form a free neutron gas. Above critical density value of $\rho = 2.8 \times 10^{17}$ kg m^−3^, the nuclei dissolve so that the protons get unclustered to move freely^9,10^. Between these two densities, the matter consists of neutron-rich-nuclei in a Coulomb lattice (strong coupling), a gas of free neutrons, and a degenerate electron gas penetrating the lattice^9^. In this regime, when the temperature is below $10^{9}$ K, the free neutrons become superfluid by forming isotropic ^1^S_0_ Cooper pairs^10,11^.

It is to be noted here that Epstein has for the first time proposed the superfluidic behaviour of neutrons in the constitutive matter of non-rotating and unmagnetized neutron stars to study their bulk acoustic instability properties^10^. The flow of neutron superfluid has been considered both around and through the constitutive nuclei. It has been found that the sound phase speed corresponding to the excited shear mode gets enhanced as the constituent neutron-rich nuclei are weakly coupled with the outer superfluidic neutrons in the inner crust region^10^. The results are applicable to the wave propagation in a neutron star as long as the perturbation wavelength is smaller than the density gradient scale lengths, also termed as spatial inhomogeneity scale lengths^10^. Besides, the main effect of the non-local gravitational field on the sound modes associated with the superfluidic matter has also been studied^12^. Two distinct sound modes have been reported to exist in the superfluid: the first sound (density wave) and the second sound (entropy wave). It has generalized the Jeans instability criteria of the sound mode in the normal fluidic counterpart^12^. It has been found that the Jeans scale length in the superfluid is ${2 / {\sqrt 3 }}$ times larger than that in the usual case of a normal fluid medium^12^.

In addition, the analysis of collective excitations of diversified waves, oscillations, and glitches in the neutron stars ensures the superfluidic behaviour of their inner crust regions. The occurrence of the spin glitches can be manifold from the viewpoint of several authors. The glitches are developed due to the sudden reorganization of the neutron star crust (by star-quake). In such models, a neutron star is a two-component structure of a superfluid core surrounded by a rigid crust^13–15^. As already reported elsewhere^3^, the glitches are due to the sudden release of the elastic energy. In such a system, a heavy nucleus is assumed to be a spherical piece of a viscoelastic Fermi-continuum compressed to the normal nuclear density. In the inner crust regime, superfluid vortices interact with the heavy nuclei and pin up with the nuclei in the Coulomb lattice as already mentioned before. The unpinning of large-scale vortices from the nuclei can also result in the form of spin glitches well observed in neutron stars^16–18^. Such glitches can also result due to the instability of vortex creeps through the nuclear lattice^8,19,20^. Only a few models have discussed that the interaction of neutron superfluid vortex filaments with the proton superconducting flux tubes in the core of the neutron star results in the evolution of glitches^21^. The glitches can also be produced because of the coupling of the crust with the superfluid inside the neutron stars^22–24^. Recently, the glitch formation is explained as a repeated phenomenon from the quasi-period ${}^{3}P_{2}$ neutron superfluid B-phase (magnetic moment of ${}^{3}P_{2}$ Cooper pairs aligned with the magnetic field) to A-phase (magnetic moments are very chaotic), and then back to B-phase repeatedly, resulting in many repeated glitches with quasi-periods^25^. But, the mechanisms operating behind the origin of such glitches from the simplistic fluidic viewpoint is yet to be illuminated as far as seen extensively in the literature.

It is to be noted here that the glitches are the potential agents to excite various collective waves and oscillation modes with different periods $(\tau )$ in neutron stars; viz., pressure (*p-*)mode, gravity (*g-*)mode, fundamental (*f-*)mode, shear (*s-*)mode, interfacial (*i-*)mode, torsional (*t*-)mode, Rossby (*r*-)mode, and gravitational wave (*w*-)mode^26^. The *p-*mode is an acoustic mode, like an ordinary sound signal, the propagation of which is dependent on the material density and temperature of the stellar media ($\tau \sim 0.1$ ms). Besides, the *g-*mode is completely confined to fluid core and caused by the buoyancy acting as a restoring force ($\tau \sim 10 - 400$ ms) and *f-*mode is a surface *g-*mode overlying the crust ($\tau \sim 0.1 - 0.8$ ms). Similarly, the *s-*mode is a normal mode of velocity shear wave present in the solid neutron star crust ($\tau \sim 1$ ms − 10 s). The *i-*mode is a hybrid pattern composed of the spectral waves propagating in the solid–fluid interfaces in the neutron star ($\tau \sim 100$ ms). The *t*-mode is the torsional motion caused by the tangential motion of the material from the neutron star surface ($\tau < \,\,20$ ms). The *r*-mode is excited in the rotating structure due to the Coriolis force acts as a restoring force along the surface ($\tau \sim 1 - 100$ ms). The *w*-mode gets generated due to the space–time curvature-induced fluctuations (fully relativistic effects). It dissipates energy through the emission of gravitational waves ($\tau \sim 1 - 10$ µs) as extensively seen in the literature^26^.

We herein perform a systematic theoretic exploration to investigate the stability effects of the inner crust properties on the local collective waves and oscillations of nuclear origin excitable in non-rotating neutron stars. An important dimension of the key motivation behind the present study is to explore the basic physical mechanism for the glitch formation from a modified multi-fluidic perspective for the first time. Accordingly, we consider, as a first step in this direction, a three-component fluid model system depicting the inner crust region of neutron stars. It consists of the viscoelastic heavy neutron-rich nuclei (strongly correlated), superfluid neutrons (uncorrelated), and degenerate quantum electrons (weakly correlated) with polytropic equations of state confined in a spherically symmetric geometry. We ignore possible relativistic effects of the constitutive electrons for the sake of pure analytic simplicity. It is physically well validated at densities below a critical density value^27^ of $10^{9}$ kg m^−3^. The normal mode sensibly supported within our bulk-fluidic model perception here is the hybrid gravito-nucleo-acoustic (GNA) instability evolving in the complex inner crust. It is the low-frequency acoustic mode excited under a unique action originating from the GNA coupling. The electrostatic influence here is caused by all the Coulombic species (electrons + nuclei) and the self-gravitational effect originates from the Newtonian species (neutrons + nuclei). The free energy for this instability is sourced in the non-zero finite driving currents (elastic, streaming) associated with the electrons and neutrons amid the constitutive nuclei as the heavy species (inertial, non-streaming). A periodic interplay between these inertia and elasticity rhythmically results in the excitation and in the subsequent propagation of the GNA waves of nuclear origin. The various modal accelerating (decelerating) and realistic stabilizing (destabilizing) agencies of the inner crust region are semi-analytically explored.

The entrainment effect induces a flow-flow coupling in the proton fluid around each neutron vortex. It generates a local magnetic field (10^14^ G) due to which the electrons scatter dissipatively^28^. Thus, the outcome is a coupling between the constitutive neutrons and the interpenetrating conglomerate of charged particles. When the timescale of the dynamical effects is shorter than the period of the oscillations of the superfluid vortices (i.e., < $10^{ - 1}$ s), the quantized superfluid vortices play no dynamical role^29^. Thus, we expect that, for a small amplitude of local oscillations, the dynamics of the vortex oscillations will not change. So, keeping in mind the above mentioned facts, we ignore the scattering of electrons via the constitutive lattice phonons and impurities sourced in the constitutive nuclei; and hence, subsequent frictional effects^30^, intrinsic dynamics of the vortices, possible pinning effects, and other vortex-vortex interactions^29^. In this paper, we ignore the superfluid flow through the nucleus via the entrainment effects. The constitutive neutrons and nuclei are coupled via the classical formalism of the non-local long-range Newtonian gravity. The collective dynamics of the dense electrons and quantum Bohm potential arising because of the inhomogeneous wave field curvature effects associated with the constitutive quantum particles is included afresh in the stability analysis of the inner crust region of the neutron stars.

### Model and formalism

We consider the inner crust region of neutron stars composed of the viscoelastic neutron-rich nuclei, neutron superfluid, and degenerate electrons in a spherically symmetric geometrical configuration relative to the centre of the entire stellar matter mass distribution. The main advantage behind considering such a symmetric model geometry lies in the simplistic but judicious reduction of the complex 3-D problem (with multiple degrees of freedom) into an equivalent simplified 1-D problem (with single degree of freedom) without any sensible loss of both generality and reliability. In other words, the non-radial complications sourced in the polar and azimuthal angular degrees of freedom are relaxed at the cost of radial symmetry of the spherical problem. The model setup includes the effects of electrostatic potential; gravitational force (due to neutrons and nuclei); thermal pressure (for nuclei); and quantum effects (degeneracy pressure and Thomas-Fermi-based Bohm potential)^31^. Here, the tiny electrons and neutrons are treated as quantum particles as their de Broglie wavelengths ($$\lambda_{dB}$$) have larger value relative to the interparticle separation distance (with super-populous de Broglie sphere, $$n\lambda_{dB}^{3} \ge 1$$)^31^. Against this de Broglie super-criticality, the constitutive heavier nuclei are considered as classical particles. As a consequence, the quantum effects (i.e., degeneracy pressure and Bohm potential) for the electrons and neutrons are taken into account^32–35^. Again, the Coulomb coupling parameter^36^ for the heavy nuclei, $$\Gamma_{Cou} = (1/4\,\pi \,\varepsilon_{0} )\{ (Z_{d} \,e)^{2} /(a\,k_{B} \,T)\}$$$$= 8 \times 10^{3}$$. Thus, $$\Gamma_{Cou} > > 1$$, implying that the nuclei are strongly coupled (crystalline). It gives rise to the viscoelastic effects responsible for both the shear mode and the bulk mode in the classical heavy nuclear fluid^37,38^. When the temperature falls below $$10^{9}$$ K, the neutrons are completely condensed into a superfluid state by forming the ^1^S_0_ Cooper pairs. The existence of such states in the inner crust of neutron stars has already been confirmed by astronomical observations of giant pulsar frequency glitches as already well detected in Vela pulsar^7,23^.

It is noteworthy further that the local fluidic oscillation period of the inner crust material ($\tau_{J} = 1.55 \times 10^{ - 3}$ s) is shorter than that of the superfluid vortex oscillation ($\tau_{v} = 10^{ - 1}$ s)^11^. It upholds the ignorance of the dynamics involved in quantized superfluid vortices in the local oscillation of the inner crust of the neutron stars. In this limit of the charged-superfluid form of the magnetohydrodynamic phase in an ordinary plasma system, the electromagnetic forces empower only the electrical charge neutrality on a bulk microscopic scale. As the adopted fluid medium is macroscopically neutral one, the presence of electromagnetic forces is ignored herewith^11^. It may be further noteworthy that the neutron star rotation is sourced in the dynamical rotation of the constitutive neutron vortices^39^. As the dynamics of such vortices play no significant role in the overall neutron star dynamics, one could ignore the rotational effects of the model neutron star without violating the generality. Moreover, $c_{s} = 6.18 \times 10^{5}$ m s^−1^ << vacuum speed of light, $c = 3 \times 10^{8}$ m s^−1^. It shows that we consider every constitutive component of the inner crust of the neutron stars to behave as non-relativistic one. So, it is judiciously expedient to consider the simplified multi-component fluid outline in such compact astroenvirons with sub-luminal fluctuations of the relevant physical variables in the current model configuration.

The evolution dynamics of the heavy viscoelastic neutron-rich nuclei fluid, neutron superfluid, degenerate electron fluid are governed by a continuity equation for flux-density conservation, momentum equation for force-density conservation, polytropic equation of states, and finally closing the system by the electro-gravitational Poisson equations. The principal goal is to analyse the normal mode (GNA instability) evolving in the complex inner crust. The electrostatic influence here is caused by the Coulombic species (electrons + nuclei) and the self-gravitational effect originates from the Newtonian species (neutrons + nuclei).

The basic governing equations of viscoelastic heavy nucleus $(h)$ fluid are continuity equation and momentum equation in spherically symmetric geometry in a coordination space $(r,t)$ with all the usual generic notations given below respectively as

1 $$\frac{{\partial \rho_{h} }}{\partial t} + \frac{1}{{r^{2} }}\frac{\partial }{\partial r}\left( {r^{2} \rho_{h} v_{h} } \right) = 0,$$

2 $$\begin{aligned} & \left[ {1 + \tau_{m} \left( {\frac{\partial }{\partial t} + v_{h} \frac{\partial }{\partial r}} \right)} \right]\left[ {\left( {\frac{\partial }{\partial t} + v_{h} \frac{\partial }{\partial r}} \right)v_{h} + \left( {\frac{{eZ_{h} }}{{m_{h} }}} \right)\frac{\partial \phi }{{\partial r}} + \frac{\partial \psi }{{\partial r}} + \left( {\frac{1}{{\rho_{h} }}} \right)\frac{{\partial P_{h} }}{\partial r}} \right] \\ & \quad = \left( {\frac{\chi }{{\rho_{h} }}} \right)\frac{1}{{r^{2} }}\frac{\partial }{\partial r}\left( {r^{2} \frac{{\partial v_{h} }}{\partial r}} \right); \\ \end{aligned}$$

here $\tau_{m}$ is the viscoelastic relaxation time^36^. $Z_{h}$, $\rho_{h}$, and $m_{h}$ are the proton number, material density, and mass, respectively, of the neutron-rich heavy nucleus. $v_{h}$ is the flow velocity of heavy nucleus fluid. $\chi = (\varsigma + ({4 \mathord{\left/ {\vphantom {4 {3)\eta }}} \right. \kern-\nulldelimiterspace} {3)\eta }})$ is the generalized effective viscosity (with shear-bulk contributions). $P_{h}$ is the thermal pressure due to the heavy nucleus^37^. $\phi$ and $\psi$ are the electrostatic and gravitational potentials developed due the charge and mass density fields, respectively.

In a similar way, the governing equations of the superfluid neutron $(n)$ with all the usual symbols are given respectively as

3 $$\frac{{\partial \rho_{n} }}{\partial t} + \frac{1}{{r^{2} }}\frac{\partial }{\partial r}\left( {r^{2} \rho_{n} v_{n} } \right) = 0,$$

4 $$\frac{{\partial v_{n} }}{\partial t} = - \frac{\partial }{\partial r}\left( {\frac{{v_{n}^{2} }}{2} + \mu_{n} + \psi } \right) + \gamma \left( {\frac{{\rlap{--} h^{2} }}{{2m_{n}^{2} }}} \right)\frac{\partial }{\partial r}\left[ {\left( {\frac{1}{{\sqrt {\rho_{n} } }}} \right)\frac{1}{{r^{2} }}\frac{\partial }{\partial r}\left( {r^{2} \frac{{\partial \sqrt {\rho_{n} } }}{\partial r}} \right)} \right];$$

here $v_{n}$ is the neutron superfluid flow speed. $\mu_{n}$ is the neutron chemical potential. $\rho_{n}$ and $m_{n}$ are the neutron material density and mass, respectively. $\gamma = (D - 2)/\left( {3D} \right)$ is a Bohmian quantum correction prefactor for the Fermions; where $D$ is the dimension of the system^31,34^. The reduced Planck constant signifying the step unit of non-local quantum action variation is given as $\rlap{--} h = 1.054 \times 10^{ - 34}$ J s. Here, in the momentum equation (Eq. 4), the convective term does not occur in the LHS. Instead, there arises a kinetic term, ${{v_{n}^{2} } \mathord{\left/ {\vphantom {{v_{n}^{2} } 2}} \right. \kern-\nulldelimiterspace} 2}$, termed here as the kinetic potential in correlation with other types of involved potential. The reason behind such terms is in the fact that the superfluid streams without viscosity with no exchange of collisional momentum with other component fluids^12,40^ in the composite fluid system adopted here.

The similar governing equations for the constitutive degenerate electron fluid $(e)$ in spherically symmetric geometry in the coordination space $(r,t)$ are respectively cast as

5 $$\frac{{\partial \rho_{e} }}{\partial t} + \frac{1}{{r^{2} }}\frac{\partial }{\partial r}\left( {r^{2} \rho_{e} v_{e} } \right) = 0,$$

6 $$\frac{{\partial v_{e} }}{\partial t} + v_{e} \frac{{\partial v_{e} }}{\partial r} = - \frac{\partial }{\partial r}\left( {\mu_{e} - \frac{e}{{m_{e} }}\phi } \right) + \gamma \left( {\frac{{\rlap{--} h^{2} }}{{2m_{e}^{2} }}} \right)\frac{\partial }{\partial r}\left[ {\left( {\frac{1}{{\sqrt {\rho_{e} } }}} \right)\frac{1}{{r^{2} }}\frac{\partial }{\partial r}\left( {r^{2} \frac{{\partial \sqrt {\rho_{e} } }}{\partial r}} \right)} \right];$$

here $v_{e}$ is the electron flow speed. $\mu_{e}$ is the electron chemical potential. $m_{e}$ is the electronic mass. $\rho_{e}$ is the electron material density of the electronic fluid. It may be noted here that the quantum effects are considered in the above, but viscous effects are ignored because of the asymptotically small $m_{e}$-value.

In our description of the considered neutron star model, Eqs. (4), (6) are the momentum balance equations of the quantum fluid of neutrons (superfluid) and of electrons (normal fluid), respectively. The last terms therein stand for their respective quantum potentials, termed originally as the de Broglie-Bohm potentials, which arise because of the inhomogeneous wave field curvature associated with these constitutive particles. In other words, the quantum potential depends on the spatial curvature of the particle wavefunction amplitude. It physically signifies the potential energy (self-energy) function of the matter wave field associated with the particles. It gives rise to the quantum trajectories followed by the quantum particles^41^. It facilitates the transference of energy from the wave field to particle and back again which accounts for energy conservation in isolated quantum system^42^. The value of quantum potential does not give (in a non-stationary quantum state) the total energy, but it represents an amount of energy in the wave field that is available to the particle at its specific position in the field. It is also found that, more pronounced the change of wave shape, the greater the amount of energy exchanged between particle and the wave field^42^. The change of shape of the wave-field is an important ingredient in determining energy transfer and storage process. These factors clearly imply that the mechanism of energy transfer and storage processes here is completely different from the corresponding classical cases and it cannot be wavefunction amplitude-dependent^42^.

A few points on the nature of the viscoelastic fluid momentum equations may be relevant in this context. As already seen conventionally, the fluid system would behave as a hyperbolic model (wave propagatory) if there is no viscosity. It would behave as a parabolic one (diffusive or dissipative) if the fluid viscosity is taken into account. It would play as an elliptic system depicting steady state or equilibrium processes if the basic conservation rules are nicely obeyed in the absence of such dissipative agents.

Against this backdrop, the generalized polytropic equation of state of the composite system describing various thermodynamical processes in a compact form^43^ is given as

7 $$P_{\alpha } = K_{\alpha } \,\rho_{\alpha }^{{\gamma_{\alpha } }} ,$$

here $K_{\alpha }$ is the polytropic constant, $\gamma_{\alpha } = (1 + n_{\alpha }^{ - 1} )$ is the polytropic exponent, and $n_{\alpha }$ is the corresponding polytropic index. The polytropic equation of state is valid for both non-relativistic and extremely relativistic limits. But, in the non-relativistic approach, $\gamma_{\alpha } = 5/3$; and in the extreme-relativistic approach, $\gamma_{\alpha } = 4/3$ with different value of $K_{\alpha }$ in both the limits^44^. In our considered model, for electrons $(\alpha = e)$ and neutrons $(\alpha = n)$, $\gamma_{\alpha } = 5/3$ with $K_{\alpha } = \left[ {2\rlap{--} h^{2} m_{\alpha }^{{ - {8 \mathord{\left/ {\vphantom {8 3}} \right. \kern-\nulldelimiterspace} 3}}} } \right]$. This represents the electron degeneracy pressure and neutron degeneracy pressure of quantum mechanical origin in the non-relativistic limit. For the classical heavy nuclei, $\alpha = h$, $K_{h} = Z_{h} k_{b} T/m_{h} = c_{s}^{2}$, and $\gamma_{h} = 1$; where, $c_{s}$ is the isothermal sound speed in the bulk fluid. In this case, the polytropic equation takes the form: $P_{h} = c_{s}^{2} \,\rho_{h}$, which is the well-known isothermal equation of state in the non-relativistic regime. It is noteworthy that $P_{\alpha }$ is not the effective system pressure. It is the pressure due to the individual constitutive species.

The electrostatic Poisson equation coupling the diverse constitutive charged species with the help of the electrostatic potential $(\phi )$ distribution sourced in their charge density fields reads as

8 $$\frac{{\partial^{2} \phi }}{{\partial r^{2} }} + \left( \frac{2}{r} \right)\frac{\partial \phi }{{\partial r}} = \frac{e}{{ \in_{0} }}\left( {\frac{{\rho_{e} }}{{m_{e} }} - \frac{{Z_{h} \,\rho_{h} }}{{m_{h} }}} \right),$$

here $\in_{0} = 8.85 \times 10^{ - 12}$ $F\,m^{ - 1}$ is the absolute permittivity of the free space (vacuum) characterizing the dense fluid exactly^45^.

Finally, we close the extreme fluid model system with the help of the self-gravitational Poisson equation relating the gravitational potential $(\psi )$ distribution with the constitutive sourced material density fields given in the customary notation^46^ as

9 $$\frac{{\partial^{2} \psi }}{{\partial r^{2} }} + \left( \frac{2}{r} \right)\frac{\partial \psi }{{\partial r}} = \,4\,\pi \,G\left( {\rho_{h} + \rho_{n} } \right),$$

here $G = 6.67 \times 10^{ - 11}$ N kg^−2^ m^2^ is the Newtonian gravitational coupling constant signifying the strength of the non-local gravitational interactions undergone by gravitating matter.

The principal goal of the presented study is to develop a theoretical model to investigate the GNA instability dynamics evolving in the complex inner crust of non-rotating neutron stars. All the relevant physical parameters $\left( F \right)$ describing the composite fluid are assumed to undergo small-scale linear perturbations $\left( {F_{1} } \right)$ relative to their corresponding hydrostatic homogeneous equilibrium values ($F_{0}$) in the presence of active geometrical curvature modulation effects (via $r^{ - 1}$). Thus, such homology perturbations grow in the harmonic form of spherical spatiotemporal waves given in the generic notations^45,47^ as

10 $$F\left( {r,t} \right) = F_{0} + F_{1} \left( \frac{1}{r} \right)\exp \left[ { - i\left( {\omega t - kr} \right)} \right],$$

11 $$F = \left[ {\rho_{i} \quad v_{ri} \quad P_{i} \quad \phi \quad \psi } \right]^{T} ,$$

12 $$F_{0} = \left[ {\rho_{i0} \quad 0\quad P_{i0} \quad 0\quad 0} \right]^{T} ,$$

13 $$F_{1} = \left[ {\rho_{i1} \quad v_{ri1} \quad P_{i1} \quad \phi_{1} \quad \psi_{1} } \right]^{T} ,$$

where $\omega$ is the angular frequency, $k$ is the angular wavenumber of the collective fluctuations and the constitutive species subscript, $i = e,\,n,\,\,h$.

Application of Eqs. (10)–(13) in Eqs. (1)–(9) transform the fluidic system to evolve in the Fourier space $$\left( {k,\omega } \right)$$ against the earlier coordination space $$(r,t)$$. Thus, the involved linear differential operators get autotransformed in the new space $$\left( {k,\omega } \right)$$ as: $${\partial \mathord{\left/ {\vphantom {\partial {\partial r}}} \right. \kern-\nulldelimiterspace} {\partial r}} \to \left( {ik - r^{ - 1} } \right)$$, $${\partial \mathord{\left/ {\vphantom {\partial {\partial t \to \left( { - i\omega } \right)}}} \right. \kern-\nulldelimiterspace} {\partial t \to \left( { - i\omega } \right)}}$$, $${{\partial^{2} } \mathord{\left/ {\vphantom {{\partial^{2} } {\partial r^{2} \to \left( { - k^{2} + 2r^{ - 2} } \right) - i\left( {2kr^{ - 1} } \right)}}} \right. \kern-\nulldelimiterspace} {\partial r^{2} \to \left( { - k^{2} + 2r^{ - 2} } \right) - i\left( {2kr^{ - 1} } \right)}}$$, and $${{\partial^{3} } \mathord{\left/ {\vphantom {{\partial^{3} } {\partial r^{3} }}} \right. \kern-\nulldelimiterspace} {\partial r^{3} }} \to$$$$\left[ {\left( {3k^{2} \,r^{ - 1} - 6r^{ - 3} } \right) + i\left( {6kr^{ - 2} - k^{3} } \right)} \right]$$. So, Eqs. (1)–(9) get Fourier-transformed respectively as

14 $$\rho_{h1} = \left[ {\left( {ik + \frac{1}{r}} \right)\frac{{\rho_{ho} }}{i\omega }} \right]\,v_{h1} ,$$

15 $$v_{h1} = \left( {ik - \frac{1}{r}} \right)\,\left[ {i\omega + \left( {k^{2} + \frac{1}{{r^{2} }}} \right)\frac{{K_{h}^{/} }}{i\omega } - \frac{{k^{2} \chi }}{{\rho_{ho} \left( {1 - i\omega \tau_{m} } \right)}}} \right]^{\,\, - 1} \,\left[ {\left( {\frac{{eZ_{h} }}{{m_{h} }}} \right)\,\phi_{1} + \psi_{1} } \right],$$

16 $$\rho_{n1} = \left[ {\left( {ik + \frac{1}{r}} \right)\frac{{\rho_{no} }}{i\omega }} \right]\,v_{n1} ,$$

17 $$v_{n1} = \left( {ik - \frac{1}{r}} \right)\,\left[ {i\omega + \frac{1}{i\omega }\left( {k^{2} + \frac{1}{{r^{2} }}} \right)\left( {K_{n}^{/} + \gamma \frac{{\rlap{--} h^{2} k^{2} }}{{4m_{n}^{2} }}} \right)} \right]^{\,\, - 1} \,\psi_{1} ,$$

18 $$\rho_{e1} = \left[ {\left( {ik + \frac{1}{r}} \right)\frac{{\rho_{eo} }}{i\omega }} \right]\,v_{e1} ,$$

19 $$v_{e1} = - \left( {ik - \frac{1}{r}} \right)\left( {\frac{e}{{m_{e} }}} \right)\,\left[ {i\omega + \frac{1}{i\omega }\left( {k^{2} + \frac{1}{{r^{2} }}} \right)\left( {K_{e}^{/} + \gamma \frac{{\rlap{--} h^{2} k^{2} }}{{4m_{e}^{2} }}} \right)} \right]^{ - 1} \,\phi_{1} ,$$

20 $$\phi_{1} = - \left( {\frac{e}{{ \in_{0} }}} \right)\,\left( {\frac{{\rho_{e1} }}{{m_{e} }} - \frac{{Z_{h} \,\rho_{h1} }}{{m_{h} }}} \right)\frac{1}{{k^{2} }},$$

21 $$\psi_{1} = \, - \left( {4\,\pi \,G} \right)\left( {\rho_{h1} + \rho_{n1} } \right)\frac{1}{{k^{2} }}.$$

This is to note further that, in obtaining Eqs. (17) and (19), we use the “Gibbs–Duhem relation”, which clearly relates the perturbed pressure with the perturbed chemical potential in the isothermal fluid condition^46^ given as

22 $$\rho_{i0} \,\partial \mu_{i1} = \partial P_{i1} .$$

Now, the perturbed pressure term after Eq. (7), as used in Eqs. (15), (17), and (19), is expressed as

23 $$P_{p1} = K_{p}^{/} \,\rho_{p1} ,$$

here $K_{p}^{/} = K_{p} \,\gamma_{p} \,\rho_{p0}^{{\gamma_{p} - 1}}$ is a new modulated polytropic constant relating the polytropic parameters with the constitutive material density of the composite fluid under consideration.

It is now clearly evident that the fluctuation dynamics of the considered neutron star model is dictated by a canonically coupled set of perturbed governing equations as enlisted in the form of Eqs. (14)–(21). In order for a simplified analysis of the complex instability, we get interested in the ultra-low frequency limit $(\omega^{a} = 0,\,\forall \,a > 1)$ of the triggered fluctuations^44^. As a result, Eqs. (14)–(21) respectively simplify canonically to

24 $$v_{h1} = i\omega \,\left( {ik - \frac{1}{r}} \right)\,\left[ {Q_{1} \,\omega + \left( {k^{2} + \frac{1}{{r^{2} }}} \right)K_{h}^{/} } \right]^{ - 1} \,\left[ {\left( {\frac{{e\,Z_{h} }}{{m_{h} }}} \right)\,\phi_{1} + \psi_{1} } \right],$$

25 $$v_{n1} = i\omega \,\left( {ik - \frac{1}{r}} \right)\,\left[ {Q_{n} + \left( {k^{2} + \frac{1}{{r^{2} }}} \right)K_{n}^{/} } \right]^{ - 1} \psi_{1} ,$$

26 $$v_{e1} = - i\omega \,\left( {ik - \frac{1}{r}} \right)\,\left( {\frac{e}{{m_{e} }}} \right)\,\left[ {Q_{e} + \left( {k^{2} + \frac{1}{{r^{2} }}} \right)\,K_{e}^{/} } \right]^{ - 1} \phi_{1} ,$$

27 $$\phi_{1} = \frac{i}{\omega }\left( {\frac{e}{{ \in_{0} }}} \right)\frac{1}{{k^{2} }}\left( {ik + \frac{1}{r}} \right)\left( {\frac{{\rho_{e0} }}{{m_{e} }}v_{e1} - \frac{{Z_{h} \,\rho_{h0} }}{{m_{h} }}v_{h1} } \right),$$

28 $$\psi_{1} = Q_{6} \,\left( {\frac{{eZ_{h} }}{{m_{h} }}} \right)\,\left( {Q_{7} \,\omega + Q_{8} - Q_{6} - Q_{2} \,Q_{3} \,\rho_{n0} } \right)^{ - 1} \phi_{1} .$$

We now apply the standard method of algebraic elimination and simplification so as to decouple Eqs. (24)–(28) into a generalized linear dispersion relation describing the ultra-low-frequency hybrid GNA instability given explicitly as

29 $$\begin{aligned} \omega = & - \left[ {Q_{3} \,Q_{11} \,\left\{ {\left( {\frac{1}{{Q_{5} + Q_{e} }}} \right)\,\left( {\frac{{\rho_{e0} }}{{m_{e} }}} \right)\,\left( {\frac{e}{{m_{e} }}} \right) + \frac{1}{{Q_{9} }}} \right\} + \left( {\frac{{Z_{h} \,\rho_{h0} }}{{m_{h} }}} \right)\left( {\frac{{e\,Z_{h} }}{{m_{h} }}} \right)\left( {Q_{3} \,Q_{6} + Q_{11} } \right)} \right] \\ & \times \left[ {\left( {\frac{{Z_{h} \rho_{h0} }}{{m_{h} }}} \right)\,\left( {\frac{{eZ_{h} }}{{m_{h} }}} \right)\,\left( {Q_{1} \,Q_{6} + Q_{10} } \right) + \left( {Q_{1} \,Q_{11} + Q_{3} \,Q_{10} } \right)\left\{ {\left( {\frac{1}{{Q_{5} + Q_{e} }}} \right)\,\left( {\frac{{\rho_{e0} }}{{m_{e} }}} \right)\,\left( {\frac{e}{{m_{e} }}} \right) + \frac{1}{{Q_{9} }}} \right\}} \right]^{ - 1} . \\ \end{aligned}$$

The various multiparametric symbols appearing in Eq. (29) are respectively given as

30 $$Q_{n} = \gamma \,\left( {k^{2} + \frac{1}{{r^{2} }}} \right)\frac{{\rlap{--} h^{2} k^{2} }}{{4m_{n}^{2} }},$$

31 $$Q_{e} = \gamma \,\left( {k^{2} + \frac{1}{{r^{2} }}} \right)\frac{{\rlap{--} h^{2} k^{2} }}{{4m_{e}^{2} }},$$

32 $$Q_{1} = - i\left[ {\left( {k^{2} + \frac{1}{{r^{2} }}} \right)\,\tau_{m} K_{h}^{/} + \left( {\frac{\chi }{{\rho_{ho} }}} \right)\,k^{2} } \right],$$

33 $$Q_{2} = \left( {4\,\pi \,G} \right)\,\left( {\frac{1}{{k^{2} }}} \right)\,\left( {k^{2} + \frac{1}{{r^{2} }}} \right),$$

34 $$Q_{3} = \left( {k^{2} + \frac{1}{{r^{2} }}} \right)\,K_{h}^{/} ,$$

35 $$Q_{4} = \left( {k^{2} + \frac{1}{{r^{2} }}} \right)\,K_{n}^{/} ,$$

36 $$Q_{5} = \left( {k^{2} + \frac{1}{{r^{2} }}} \right)\,K_{e}^{/} ,$$

37 $$Q_{6} = Q_{2} \,\left( {Q_{4} + Q_{n} } \right)\,\rho_{n0} ,$$

38 $$Q_{7} = Q_{1} \left( {Q_{4} + Q_{n} - Q_{2} \rho_{n0} } \right),$$

39 $$Q_{8} = Q_{3} \,\left( {Q_{4} + Q_{n} } \right),$$

40 $$Q_{9} = \left( {\frac{e}{{ \in_{0} }}} \right)\,\frac{1}{{k^{2} }}\,\left( {k^{2} + \frac{1}{{r^{2} }}} \right),$$

41 $$Q_{10} = Q_{1} \,\left( {Q_{8} - Q_{6} - Q_{2} \,Q_{3} \,\rho_{n0} } \right) + Q_{3} \,Q_{7} ,$$

42 $$Q_{11} = Q_{1} \,\left( {Q_{8} - Q_{6} - Q_{2} \,Q_{3} \,\rho_{n0} } \right).$$

In order for executing a scale-invariant analysis, a standard astronomical normalization scheme^45,47^ is adopted to normalize Eq. (29) as

43 $$\begin{aligned} \Omega = & - \frac{1}{{\omega_{J} }}\left[ {K_{J}^{2} \,Q_{3}^{*} \,Q_{11}^{*} \left\{ {\left( {\frac{1}{{K_{J}^{2} \,Q_{5}^{*} + K_{J}^{4} \,Q_{e}^{*} }}} \right)\left( {\frac{{e\,\rho_{e0} }}{{m_{e}^{2} }}} \right) + \frac{1}{{Q_{9}^{*} }}} \right\} + e\,\rho_{h0} \left( {\frac{{Z_{h} }}{{m_{h} }}} \right)^{2} \left( {K_{J}^{2} \,Q_{3}^{*} \,Q_{6}^{*} + Q_{11}^{*} } \right)} \right] \\ & \times \left[ {e\,\rho_{h0} \left( {\frac{{Z_{h} }}{{m_{h} }}} \right)^{2} \left( {K_{J}^{2} \,Q_{1}^{*} \,Q_{6}^{*} + Q_{10}^{*} } \right) + K_{J}^{2} \left( {Q_{1}^{*} \,Q_{11}^{*} + Q_{3}^{*} \,Q_{10}^{*} } \right)\left\{ {\left( {\frac{1}{{K_{J}^{2} \,Q_{5}^{*} + K_{J}^{4} \,Q_{e}^{*} }}} \right)\,\left( {\frac{{e\,\rho_{e0} }}{{m_{e}^{2} }}} \right) + \frac{1}{{Q_{9}^{*} }}} \right\}} \right]^{ - 1}, \\ \end{aligned}$$

here $\omega_{J} = (4\pi \,G\rho_{h0} )^{{{1 \mathord{\left/ {\vphantom {1 2}} \right. \kern-\nulldelimiterspace} 2}}}$ is the Jeans frequency corresponding to constitutive heavy nuclei. $\Omega = \omega /\omega_{J}$ is the Jeans-normalized fluctuation frequency. The Jeans-normalized radial distance and wavenumber are $\xi = r/\lambda_{J}$ and $K = k/k_{J}$, respectively. The values of the Jeans angular frequency, $\omega_{J} = 6.48 \times 10^{2}$ s^−1^, the Jeans time, $\tau_{J} = 1.55 \times 10^{ - 3}$ s $\sim 1$ ms, the Jeans wavenumber $k_{J} \sim 1$ mm^−1^, the Jeans wavelength, $\lambda_{J} \sim 10^{3}$ m, and $c_{s} \sim 10^{5}$ m s^−1^.

The resulting various symbols of physical relevance, appearing in Eqs. (30)–(42) for the fluctuation dynamics get accordingly auto-normalized, respectively presented as

44 $$Q_{n}^{*} = \gamma \,\left( {K^{2} + \frac{1}{{\xi^{2} }}} \right)\frac{{\rlap{--} h^{2} K^{2} }}{{4m_{n}^{2} }},$$

45 $$Q_{e}^{*} = \gamma \,\left( {K^{2} + \frac{1}{{\xi^{2} }}} \right)\frac{{\rlap{--} h^{2} K^{2} }}{{4m_{e}^{2} }},$$

46 $$Q_{1}^{*} = - i\,\left[ {\left( {K^{2} + \frac{1}{{\xi^{2} }}} \right)\,\tau_{m} K_{h}^{/} + \frac{{K^{2} \chi }}{{\rho_{ho} }}} \right],$$

47 $$Q_{2}^{*} = \left( {4\,\pi \,G} \right)\frac{1}{{K^{2} }}\left( {K^{2} + \frac{1}{{\xi^{2} }}} \right),$$

48 $$Q_{3}^{*} = \left( {K^{2} + \frac{1}{{\xi^{2} }}} \right)\,K_{h}^{/} ,$$

49 $$Q_{4}^{*} = \left( {K^{2} + \frac{1}{{\xi^{2} }}} \right)\,K_{n}^{/} ,$$

50 $$Q_{5}^{*} = \left( {K^{2} + \frac{1}{{\xi^{2} }}} \right)\,K_{e}^{/} ,$$

51 $$Q_{6}^{*} = Q_{2}^{*} \left( {K_{J}^{2} \,Q_{4}^{*} + K_{J}^{4} \,Q_{n}^{*} } \right)\,\rho_{n0} ,$$

52 $$Q_{7}^{*} = K_{J}^{2} \,Q_{1}^{*} \left( {K_{J}^{2} \,Q_{4}^{*} + K_{J}^{4} \,Q_{n}^{*} - Q_{2}^{*} \,\rho_{n0} } \right),$$

53 $$Q_{8}^{*} = K_{J}^{2} \,Q_{3}^{*} \left( {K_{J}^{2} \,Q_{4}^{*} + K_{J}^{4} \,Q_{n}^{*} } \right),$$

54 $$Q_{9}^{*} = \left( {\frac{e}{{ \in_{0} }}} \right)\frac{1}{{K^{2} }}\left( {K^{2} + \frac{1}{{\xi^{2} }}} \right),$$

55 $$Q_{10}^{*} = K_{J}^{2} \,Q_{1}^{*} \left( {Q_{8}^{*} - Q_{6}^{*} - K_{J}^{2} \,Q_{2}^{*} \,Q_{3}^{*} \,\rho_{n0} } \right) + K_{J}^{2} \,Q_{3}^{*} \,Q_{7}^{*} ,$$

56 $$Q_{11}^{*} = K_{J}^{2} \,Q_{1}^{*} \left( {Q_{8}^{*} - Q_{6}^{*} - K_{J}^{2} \,Q_{2}^{*} \,Q_{3}^{*} \,\rho_{n0} } \right).$$

It is clearly evident that the dispersion properties of the low-frequency GNA fluctuations (governed by Eq. 43) excited in the inner crust region of neutron stars are basically dictated by the multiparametric dispersion windows featuring the interior of neutron stars (described judiciously by Eqs. 44–56).

## Results and discussions

**Figure 1:**
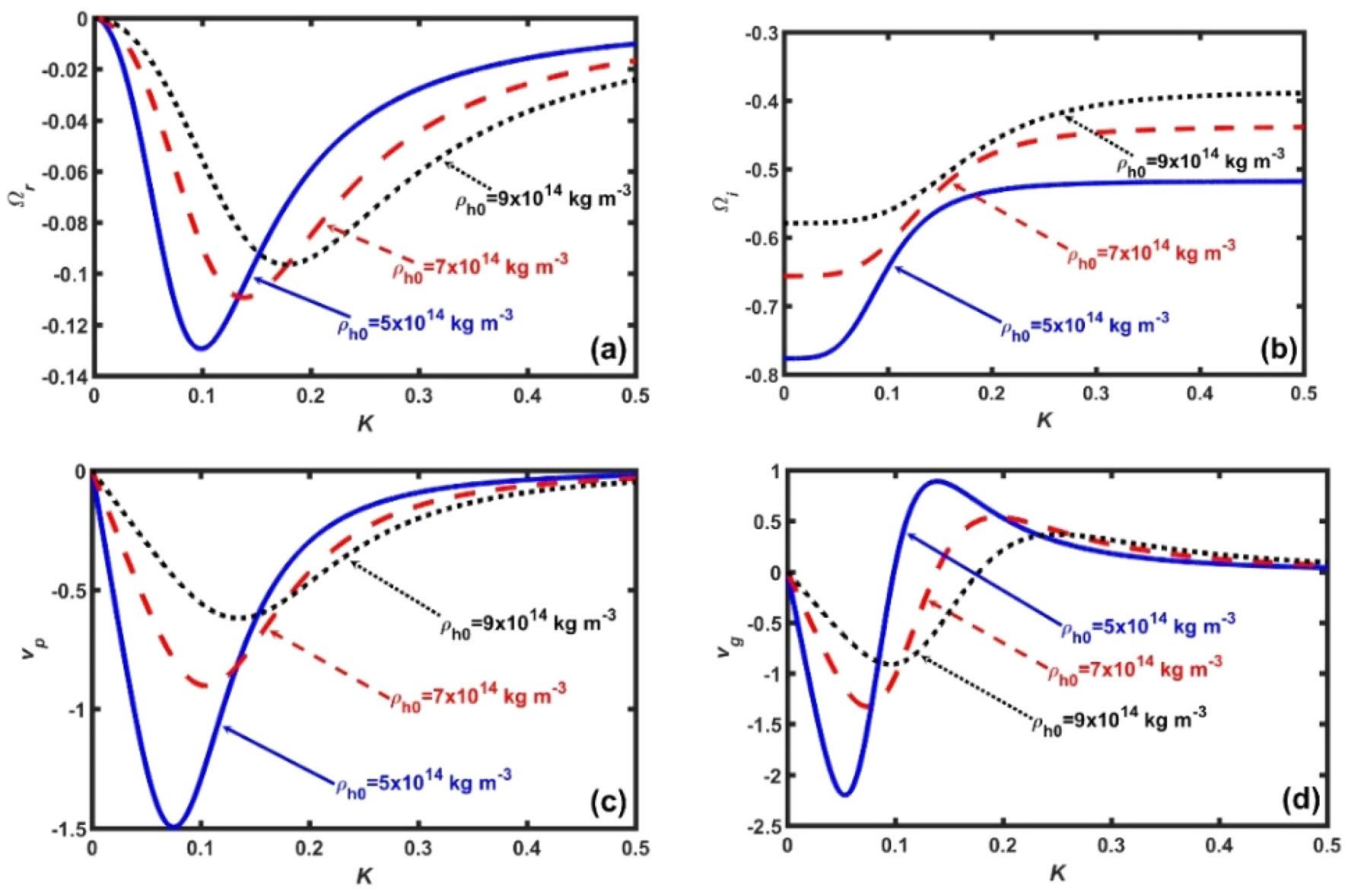
Profile of the Jeans-normalized (**a**) real frequency $(\Omega_{r} )$, (**b**) imaginary frequency $(\Omega_{i} )$, (**c**) phase velocity $(v_{p} )$, and (**d**) group velocity $(v_{g} )$ of the fluctuations with variation in the Jeans-normalized wavenumber $(K)$ for the different $\rho_{h0}$-values. The distinct lines link to $\rho_{h0} = 5 \times 10^{14}$ kg m^−3^ (solid blue line), $\rho_{h0} = 7 \times 10^{14}$ kg m^−3^ (red dash-dash line), and $\rho_{h0} = 9 \times 10^{14}$ kg m^−3^ K (black dotted line), respectively.

**Figure 2:**
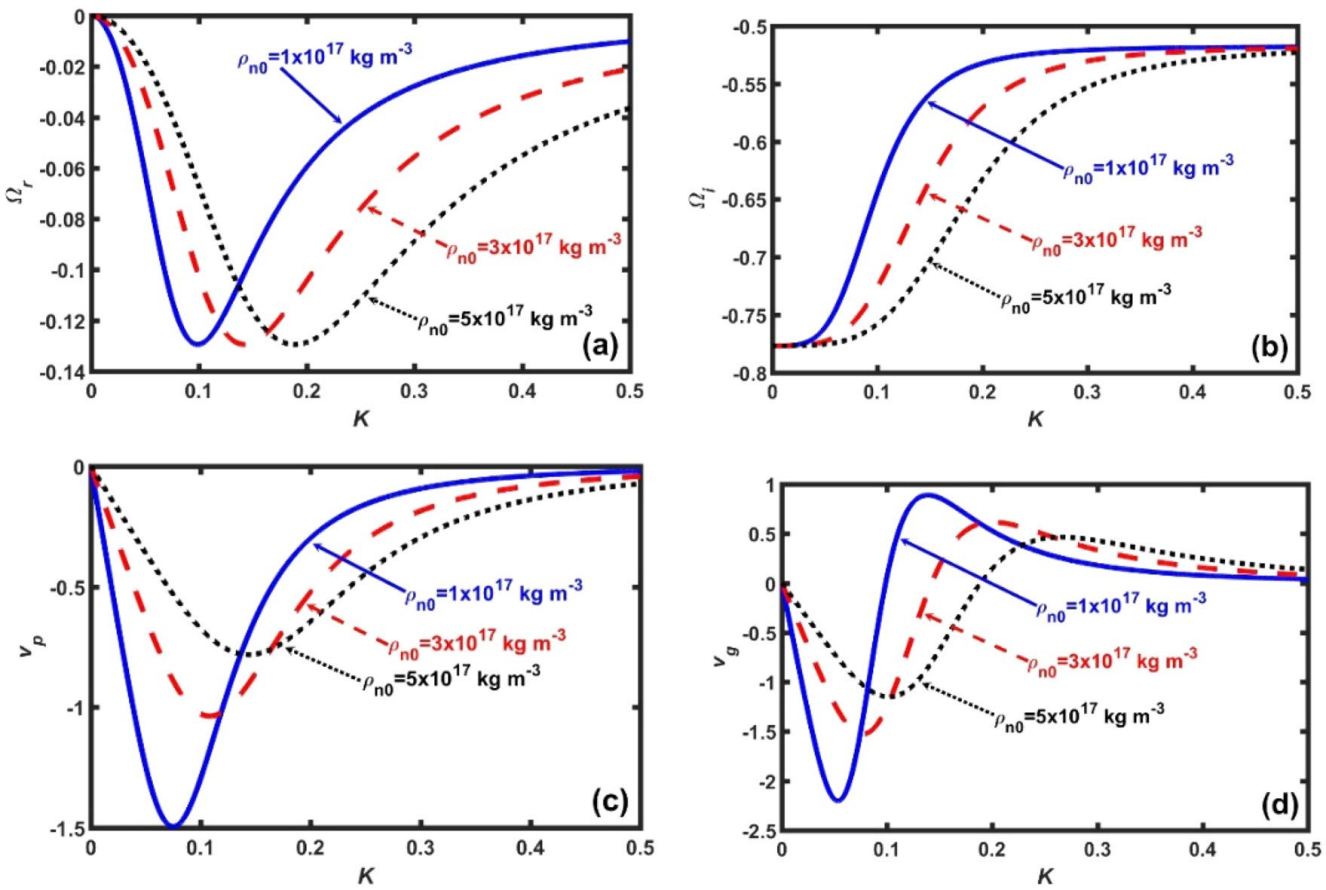
Same as Fig. 1, but for $\rho_{h0} = 5 \times 10^{14}$ kg m^−3^ (fixed). The different lines link to $\rho_{n0} = 1 \times 10^{17}$ kg m^−3^ (solid blue line), $\rho_{n0} = 2 \times 10^{17}$ kg m^−3^ (red dash-dash line), and $\rho_{n0} = 3 \times 10^{17}$ kg m^−3^ (black dotted line), respectively.

**Figure 3:**
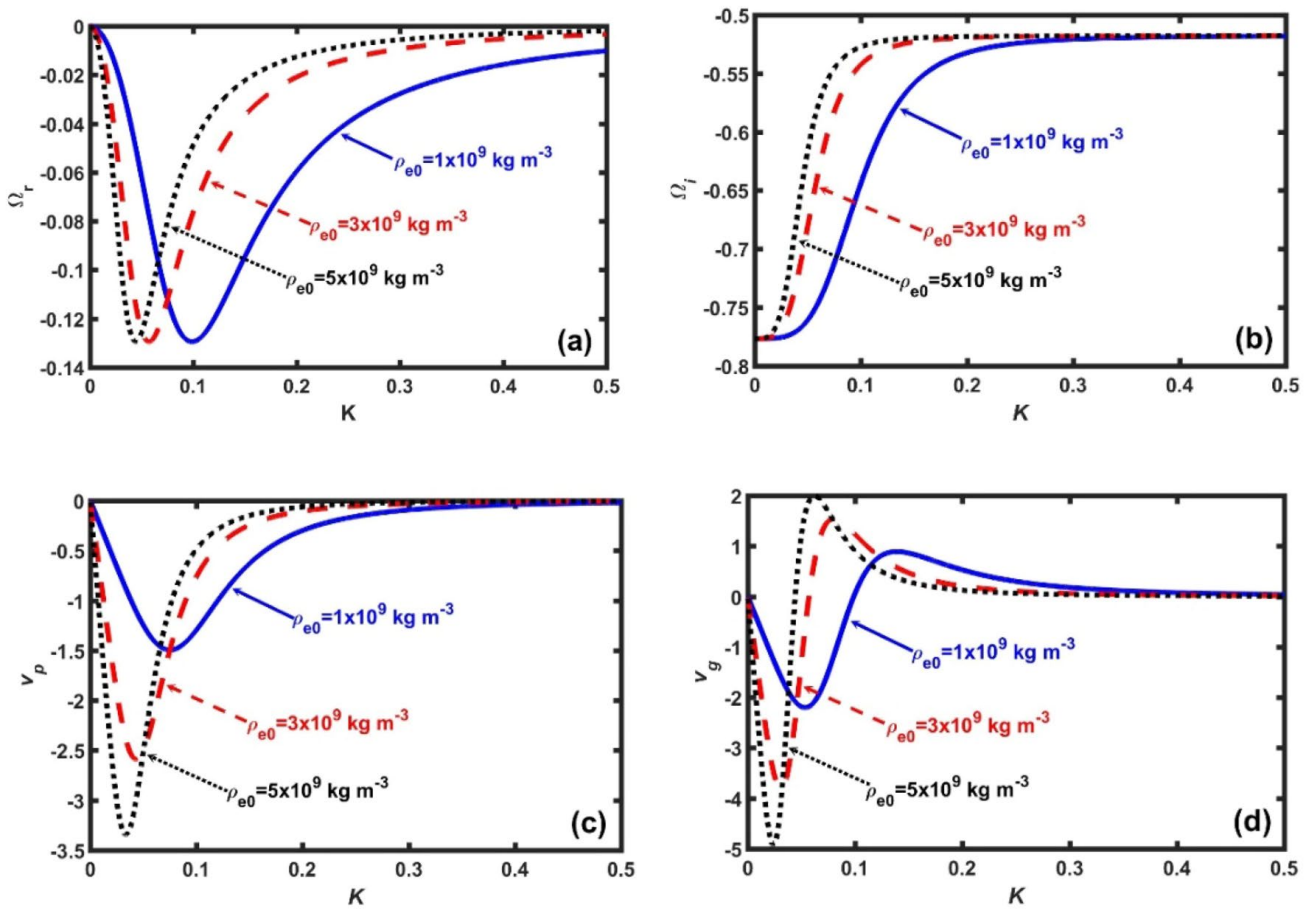
Same as Fig. 1, but for $\rho_{h0} = 1 \times 10^{15}$ kg m^−3^ (fixed). The different lines link to $\rho_{e0} = 1 \times 10^{9}$ kg m^−3^ (solid blue line), $\rho_{e0} = 2 \times 10^{19}$ kg m^−3^ (red dash-dash line), and $\rho_{e0} = 3 \times 10^{9}$ kg m^−3^ (black dotted line), respectively.

**Figure 4:**
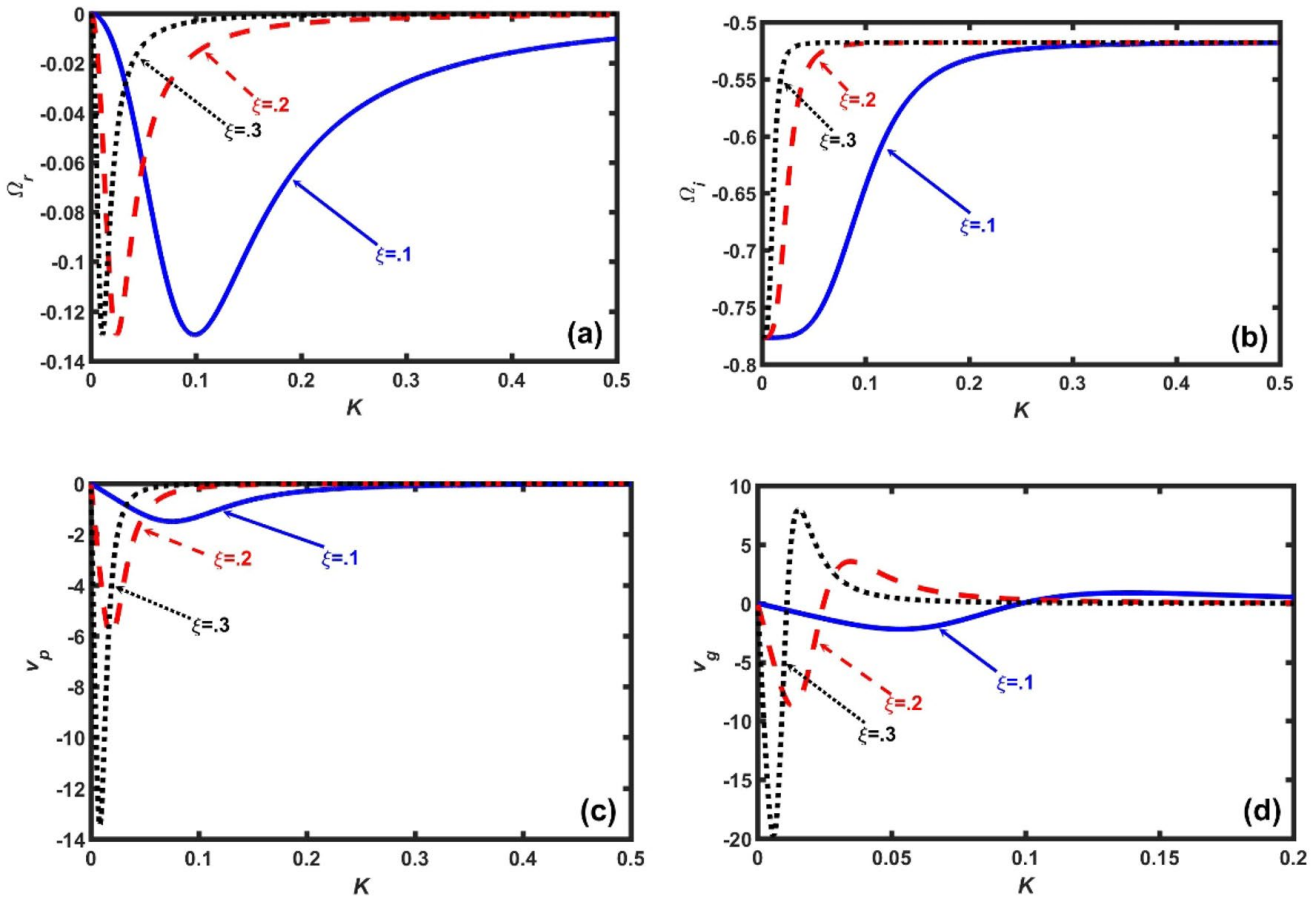
Same as Fig. 1, but for $\rho_{h0} = 5 \times 10^{14}$ kg m^−3^ (fixed). The different lines link to different Jeans-normalized radial space coordinates as $\xi = 0.1$ (solid blue line), $\xi = 0.2$ (red dash-dash line), and $\xi = 0.3$ (black dotted line), respectively.

**Figure 5:**
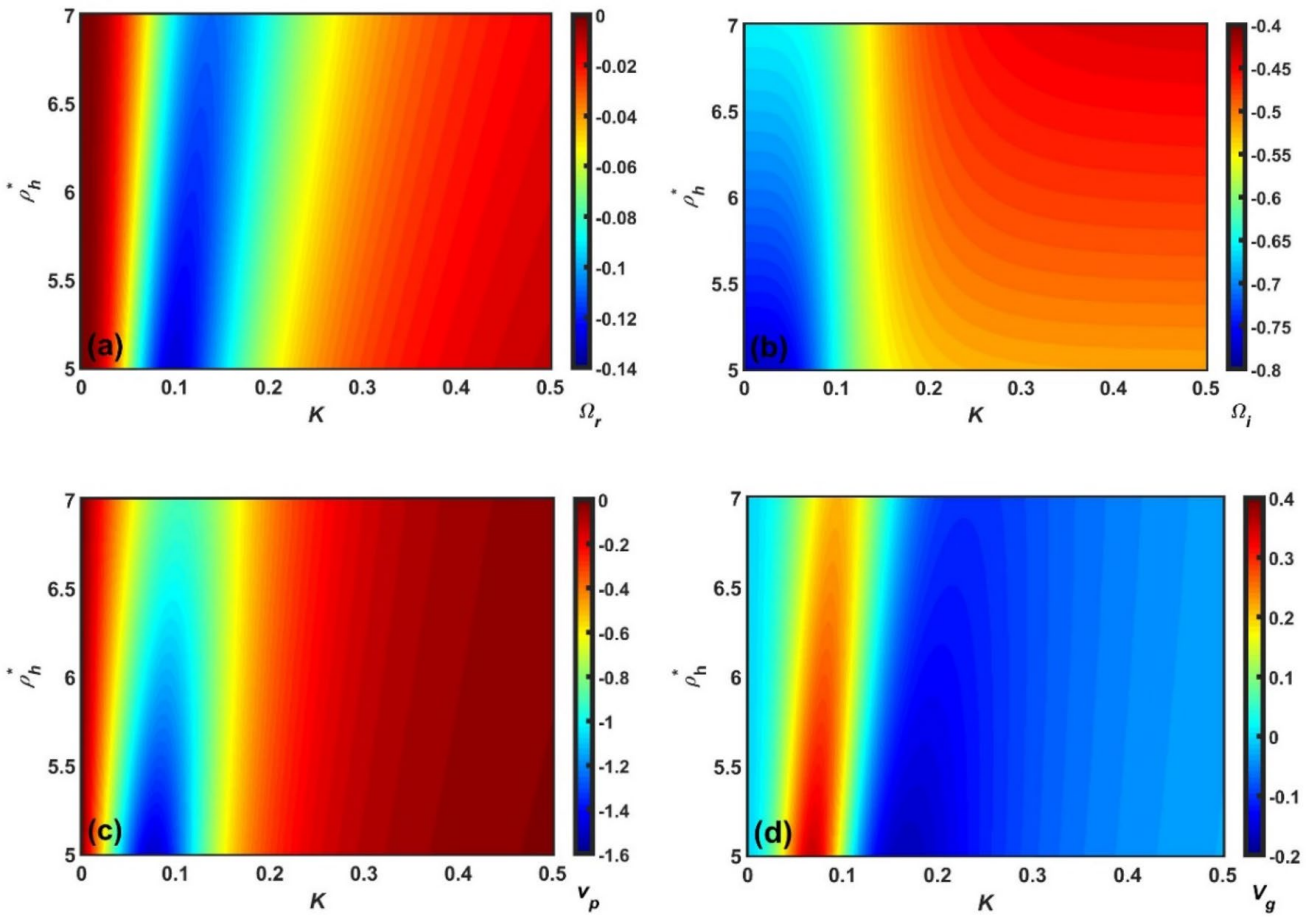
Spectral profile of the Jeans-normalized (**a**) real frequency $(\Omega_{r} )$, (**b**) imaginary frequency $(\Omega_{i} )$, (**c**) phase velocity $(v_{p} )$, and (**d**) group velocity $(v_{g} )$ of the GNA fluctuations in a colour phase space functionally defined by the Jeans-normalized angular wavenumber $(K)$ and the rescaled heavy nuclear material density $(\rho_{h}^{*} )$.

**Figure 6:**
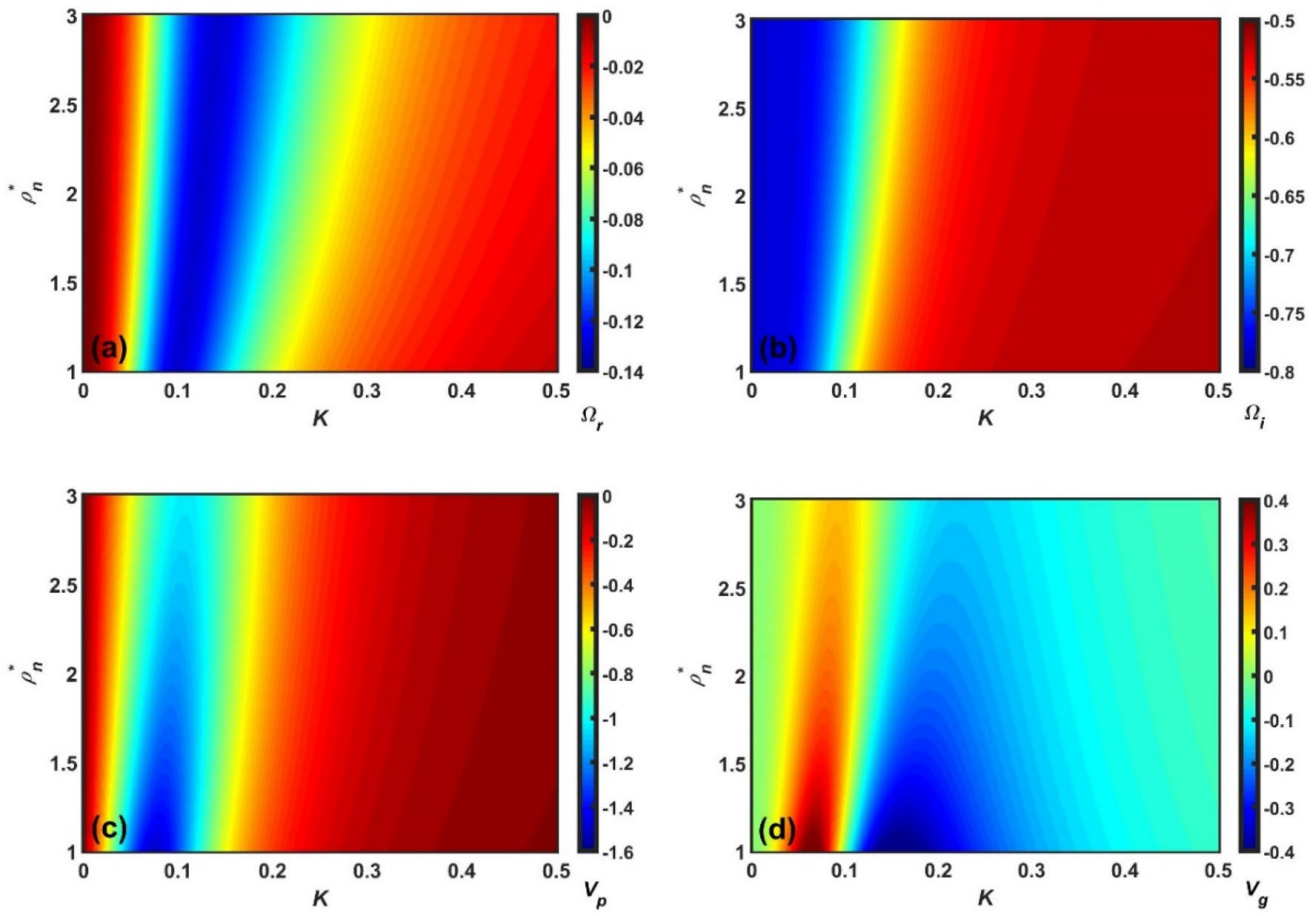
Same as Fig. 5, but showing the $\rho_{n}^{*}$-variation with a fixed $\rho_{h}^{*}$.

**Figure 7:**
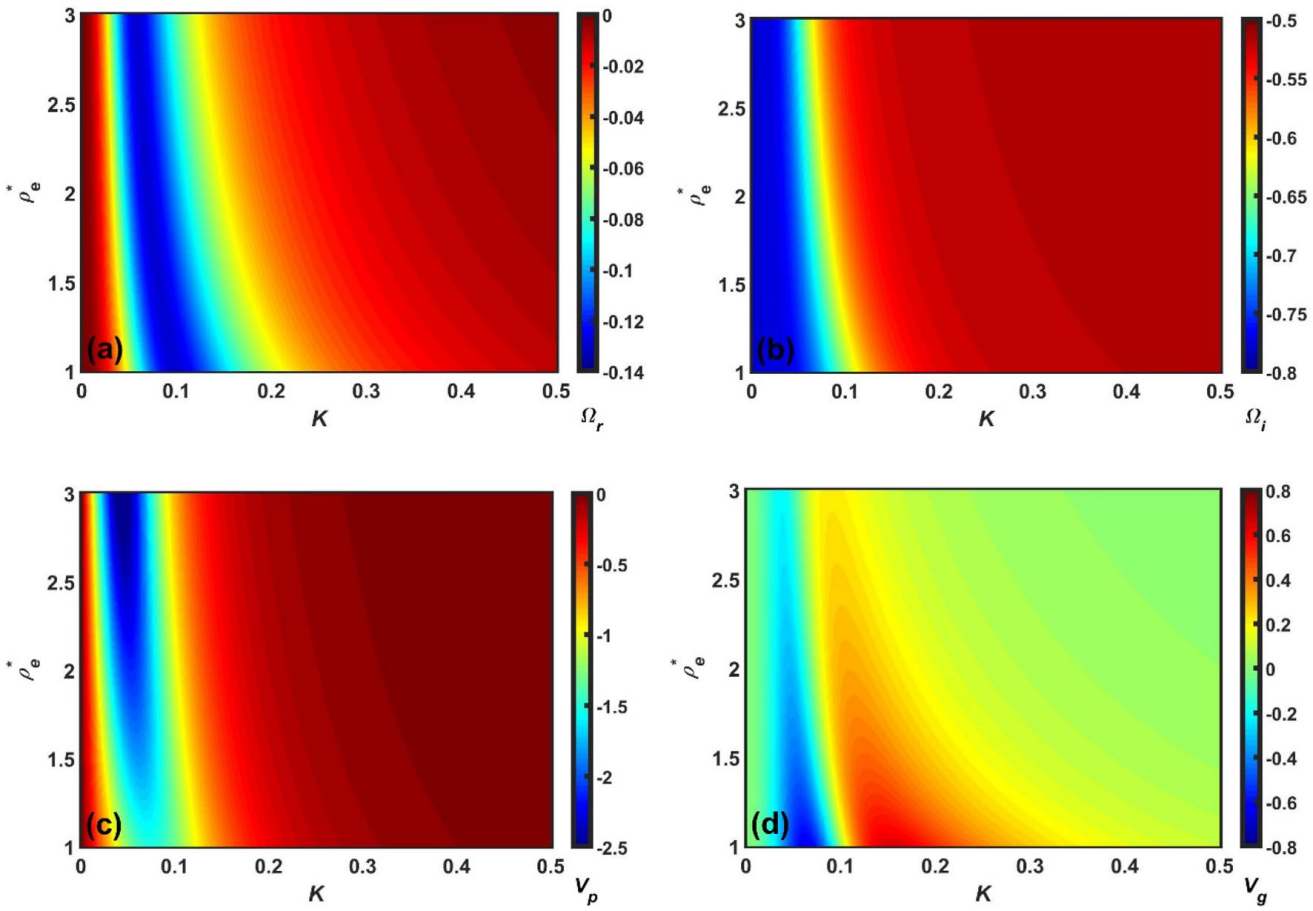
Same as Fig. 5, but showing the $\rho_{e}^{*}$-variation with a fixed $\rho_{h}^{*}$.

**Figure 8:**
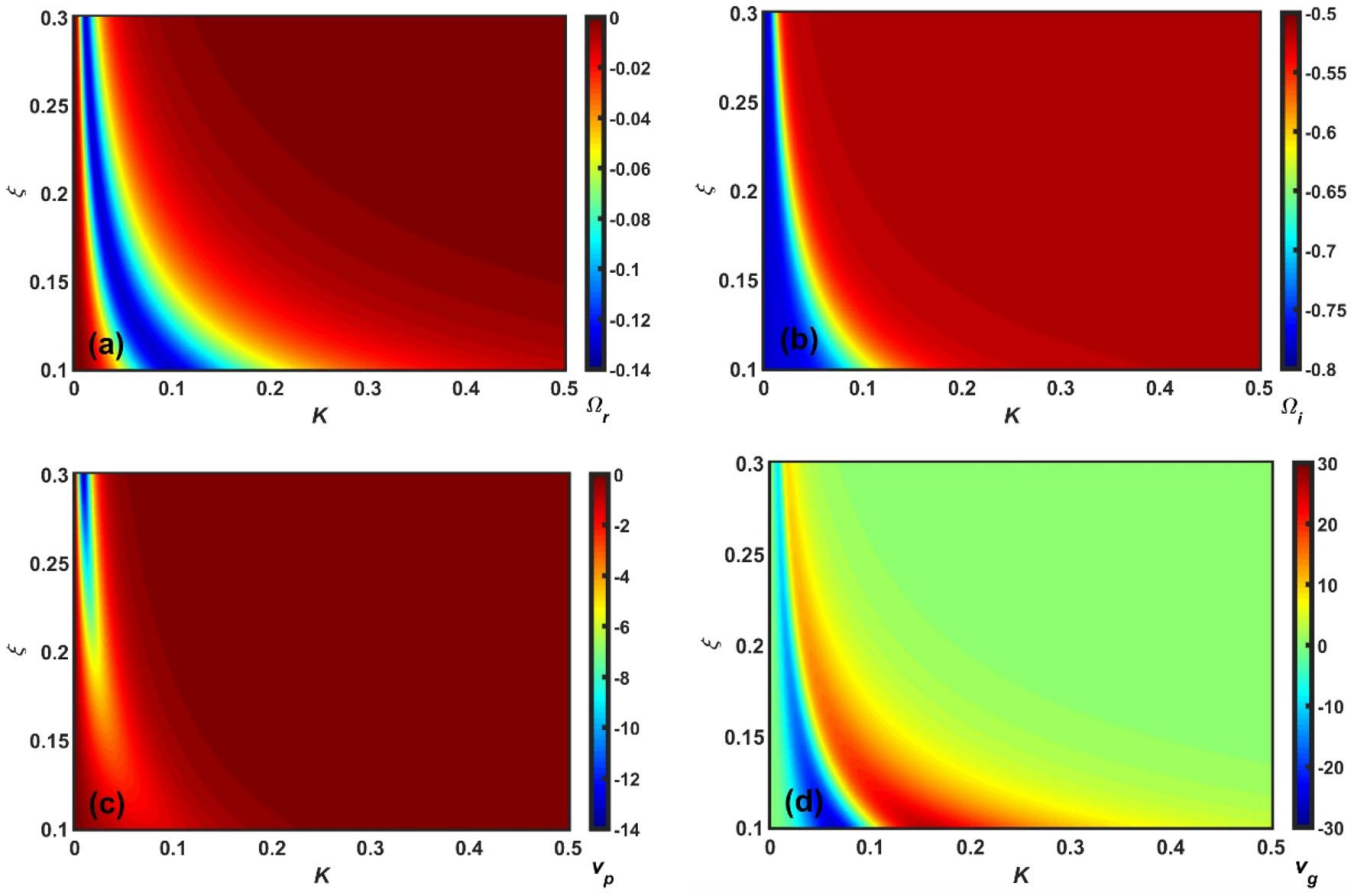
Same as Fig. 5, but showing the $\xi$-variation with a fixed $\rho_{h}^{*}$.

In the proposed semi-analytic work, we study the collective excitation of radial waves and oscillations in the inner crust region of neutron stars in the strategic framework of generalized hydrodynamic model in an assumed spherically symmetric geometry. The inner crust is composed of degenerate electrons, superfluid neutrons, and heavy neutron–rich nuclei inconclusively coupled via the gravito-electrostatic Poisson formalism. The small-amplitude spherical normal mode analysis yields a linear dispersion relation (Eq. 43), modulated by an atypical set of coefficients (Eqs. 44–56), multiparametrically dependent on the diversified inner crust features. It is numerically analysed to explore the various instability properties (Figs. 1, 2, 3, 4, 5, 6, 7 and 8). The various reliable inputs^10,11,27,34,48^ used herein are: $m_{e} = 9.1 \times 10^{ - 31}$ kg, $m_{n} = 1.67 \times 10^{ - 27}$ kg, $m_{h} = 1.3 \times 10^{ - 25}$ kg, $\rho_{e0} = 1 \times 10^{9}$ kg m^−3^, $\rho_{n0} = 1 \times 10^{17}$ kg m^−3^, $\rho_{h0} = 5 \times 10^{14}$ kg m^−3^, $Z_{h} = 36$, $T = 10^{8}$ K, $\tau_{m} = 10^{ - 3}$ s, $\chi = 10^{10}$ kg m^−1^ s^−1^, and $\gamma = - 1/3$.

In Fig. 1, we show the Jeans-normalized (a) Real frequency $(\Omega_{r} )$, (b) Imaginary frequency $(\Omega_{i} )$, (c) Phase velocity $(v_{p} )$, and (d) Group velocity $(v_{g} )$ of the fluctuations in the reciprocal wave space defined by the Jeans-normalized wavenumber $(K)$. The distinct lines herein link to $\rho_{h0} = 5 \times 10^{14}$ kg m^−3^ (blue solid line), $\rho_{h0} = 7 \times 10^{14}$ kg m^−3^ (red dashed line), and $\rho_{h0} = 9 \times 10^{14}$ kg m^−3^ (black dotted line), respectively. The normalized heavy nucleus density is herewith scaled down as $\rho_{h}^{*} = \rho_{h0} \times 10^{ - 14} = n_{h0} m_{h} \times 10^{ - 14}$ with the rescaling factor taken to be $10^{ - 14}$; where, $n_{j0}$ is the equilibrium concentration of the species-*j*, with $j = e,\,n,\,h$. It is seen that, with increase in $\rho_{h}^{*}$, $\Omega_{r}$ increases and shifts towards the high-*K* regime, and vice-versa (Fig. 1a). We further see that the fluctuations are highly dispersive in nature in the quasi-acoustic domain against the gravitational one. It means that the short-wavelength acoustic mode are excited in the high-*K* regime, and vice-versa. Again, it is seen that, both the $\Omega_{r}$-value (Fig. 1a) and the $\Omega_{i}$-value (Fig. 1b) increase with increase in $\rho_{h}^{*}$, and vice-versa. This implies that with the increase in $\rho_{h}^{*}$, the inward gravitational force increases, weakening the radially outward non-gravitational counter-force. It results in an enhancement of the harmonic oscillations executed by the inner crust region; thereby, finally, leading to the inner crust collapse if there exists no fuel to counter the inward self-gravity. It is seen that the minimum decay separation corresponding to the $\rho_{h}^{*}$-variation occurs in a narrow-*K* region at around $K = 0.17$ (Fig. 1b). Both before and after this *K*-region, the wave decay rates are flattened in the *K*-space. It is further seen that, both $v_{p}$ (Fig. 1c) and $v_{g}$ (Fig. 1d) decrease with $\rho_{h}^{*}$, and vice-versa. The negative value of $v_{p}$ (Fig. 1c) implies that the wave is propagating towards the centre of the neutron star core. It is attributed that the decrease in both $v_{p}$ (Fig. 1c) and $v_{g}$ (Fig. 1d) is due to an enhanced viscosity of the constituent heavy nuclear matter fluid. It hereby implies that the acoustic wave fluctuations slow down as this move radially inward to a stability point in the inner crust region. This result is contrary to that obtained by Epstein, where the sound phase speed is enhanced as the neutron rich nuclei are weakly coupled to the neutron in the crust^10^. The difference is due to the Epstein consideration of the neutron superfluid flow through the constitutive nuclei. In contrast, we consider that the constitutive neutron and heavy nuclear fluids are coupled via the long-range non-local gravitational force. The GNA mode propagation is indeed a two-step process in the monochromatic picture (Fig. 1c). It means that the bulk mode under consideration behaves as a dispersive *g-*mode in the $K$-space defined by $0 < K < 0.5$. Beyond this, the mode undergoes a quasi-linear transformation into a non-dispersive acoustic *p-*mode in the $K$-space. It is evident now that the bulk mode propagation is a three-step process in the polychromatic wave-packet modal picture (Fig. 1d) against the previous monochromatic portrayal (Fig. 1c). It means that the modal spectral components move inward in a dispersive fashion in the $K$-space defined by $0 < K < 0.1$. After this limit, the bulk mode moves radially outward in a quasi-dispersive manner. A close comparison between the velocity profiles allows us to draw a common inference that $\rho_{h}^{*}$ acts as a deceleration agency to the propagatory GNA mode. At the same time, a single monochromatic pulse and its equivalent group counterpart significantly differ in terms of the propagatory features (Fig. 1c–d). The basic physics behind is in the incoherent phase and amplitude coordination among the background constitutive spectral components (via coherence and decoherence). It allows us to infer that $\rho_{h}^{*}$ acts as a decelerating and destabilizing agency to the considered fluctuations towards the neutron star core.

In Fig. 2, we display the same as Fig. 1, but for a fixed $\rho_{h0} = 5 \times 10^{14}$ kg m^−3^ and for different rescaled values of $\rho_{n}^{*} = \rho_{n0} \times 10^{ - 17} = n_{n0} \,m_{n} \times 10^{ - 17}$. Here, the density rescaling factor, $10^{ - 17}$, is used to produce smooth profiles. It is seen that, as $\rho_{n}^{*}$ increases, the magnitude of the $\Omega_{r}$-peak remains unchanged; but, only gets shifted towards the high-*K* regime (Fig. 2a). It implies that, the hybrid GNA waves get highly dispersive with enhanced $\rho_{n}^{*}$. It hereby implicates that the short-wavelength acoustic modes are excited against the inhomogeneous gravity-induced modes. Thus, it supports the fact that superfluidic modes are predominately acoustic in nature and the superfluidity prevents the *g*-modes to behave pulsationally^49^. In other words, the non-local gravito-acoustic coupling is significantly opposed, thereby, resulting in the non-pulsating *g*-modes as a new natural phenomenology. As a result, it can be herewith inferred that $\rho_{n}^{*}$ plays as a dispersive broadening agency to the GNA mode. It is found further that, as $\rho_{n}^{*}$ increases, $\Omega_{i}$ decreases in the particular *K*-range defined by $0.05 \le K \le 0.45$ (Fig. 2b). It means that the neutron degeneracy pressure increases with $\rho_{n}^{*}$; thereby, opposing the inward pull caused by the non-local gravitational and Bohm potentials. The inward core-centric direction of the quantum mechanical Bohm potential is due to $\rho_{n}^{*} > 0$; thus, making the Bohm potential negative^41^. As a result, the curvature of the neutron density modulus is upward in this classically forbidden region and wave function is decreasing rapidly. It is further speculative that the maximum decay separation corresponding to the $\rho_{n}^{*}$-variation occurs in a short-*K* regime around $K = 0.17$ (Fig. 2b). It is attributable to the high sensitivity of the neutron degeneracy pressure of non-gravitational origin against the quasi-linear coupling of the gravito-acoustic triggering effects. In addition, the patterns of $v_{p}$ (Fig. 2c) and $v_{g}$ (Fig. 2d) vary similarly as before (Fig. 1c–d). Thus, $\rho_{n}^{*}$ introduces a decelerating and stabilizing influence to the said instability in the range $0.05 \le K \le 0.45$.

In Fig. 3, we portray the same as Fig. 1, but for a fixed $\rho_{h0} = 5 \times 10^{14}$ kg m^−3^ and for different rescaled values of $\rho_{e}^{*} = \rho_{e0} \times 10^{ - 9} = n_{e0} \,m_{e} \times 10^{ - 9}$. Here, the density rescaling factor is $10^{ - 9}$ for smooth variations. It is seen further that, with increase in $\rho_{e}^{*}$, the magnitude of the $\Omega_{r}$-peak value does not change; but, it shifts towards the low-*K* value, and vice-versa (Fig. 3a). It implies that, in the inner crust region only electrons facilitate the long-wavelength gravitational fluctuations $(K \to 0)$ to undergo resonance growth on the grounds of atypical gravito-electrostatic interplay mechanism. As a result, it can be herewith inferred that $\rho_{e}^{*}$ plays as an anti-dispersive narrowing agency. In contrast, the $\Omega_{i}$-value increases in the *K*-range defined by $0 \le K \le 0.3$ (Fig. 3b). It implicates that, an enhancement in the electronic concentration intensifies the electron degeneracy pressure, thereby, reducing the neutron degeneracy pressure. This situation is realizable if negative beta decay $(n \to p + e^{ - } + \overline{\nu }_{e} )$ occur in the regime $0 \le K \le 0.3$. As a result, anti-neutrinos are emitted from this regime of the neutron stars. It is further noticed that the maximum decay separation in the *K*-space corresponding to the $\rho_{e}^{*}$-variation occurs in a short-*K* regime around $K = 0.07$ (Fig. 3b). It is attributable to the high (low) sensitivity of the electron (neutron) degeneracy pressure of non-gravitational origin against the quasi-linear coupling of the gravito-acoustic triggering effects. The $v_{p}$-patterns (Fig. 3c and $v_{g}$-patterns Fig. 3d) are just reversed with respect to the previous cases (Fig. 2c–d), but now with higher respective magnitudes. It indicates that $\rho_{e}^{*}$ acts as a speeding-up factor for the waves travelling core-wards of neutron stars. That is to say, interestingly, that $\rho_{e}^{*}$ acts as an accelerating and destabilizing agent to the collective hybrid GNA instability dynamics in the inner crust region of neutron stars.

As in Fig. 4, we portray the same as Fig. 1, but for a fixed $\rho_{h0} = 5 \times 10^{14}$ kg m^−3^ and for different values of $\xi$. It is seen that the instability spectral patterns vary in a correlative and similar consistent fashion with increase in $\xi$, as in Fig. 3, with arrangement in $\rho_{e}^{*}$. It means that, as the geometrical curvature of the inner crust region increases, the magnitude of the Bohm potential increases core-wards. As a result, the resultant inward pressure force overcomes the resultant pressure counterpart. It is further noted that the maximum decay separation in the *K*-space corresponding to the $\xi$-variation occurs in a short-*K* regime at around $K = 0.05$ (Fig. 4b). Thus, it can be conjectured that $\xi$ acts as an accelerating destabilizer to the GNA fluctuations.

Clearly, Figs. 5, 6, 7 and 8 depict the same as Figs. 1, 2, 3 and 4, but in a more precise way describing the variation of $\Omega_{r}$, $\Omega_{i}$, $v_{p}$, and $v_{g}$ with *K* using a colour spectral analysis in a defined colour phase space. The blue and red represent the least and most effectiveness of the parameter of concern in a particular regime of $K$, respectively. A common instability feature found in Figs. 5, 6, 7 and 8 is that $\Omega_{r}$, $\Omega_{i}$, and $v_{p}$ are strongly dominated in the high-$K$ regime; whereas, $v_{g}$ is in the low-$K$ regime. In addition, it is interesting to note that the high-$K$ regime is the most unstable zone indicating the fact that acoustic mode plays a dominant role in the outer inner crust regime. All other features are very similar to the corresponding line profile depictions (Figs. 1, 2, 3 and 4). As a result, it could herewith be conjectured that the scale invariance of the basic physical insights behind the GNA instability features could be established in the compact astroenvirons of the neutron star family.

## Conclusions

We propose a theoretic generalized model development describing a three-component semi-analytic formalism to investigate the modal stability behaviours of the inner crust properties of non-rotating neutron stars in terms of the locally excitable collective GNA instability waves and oscillations. The adopted model consists of viscoelastic heavy neutron-rich nuclei, superfluid neutrons, and degenerate quantum electrons treated in a spherically symmetric geometry. The assumed symmetric geometry transforms the complex 3-D spherical problem into the corresponding simple 1-D radial problem free from the polar and azimuthal degrees of freedom. A normal spherical mode analysis yields a generalized linear dispersion relation, which has a unique set of dispersion coefficients, multiparametrically dependent on the inner crust features of neutron stars. It principally aims to analyse the most relevantly supported normal mode, the GNA modal wave and associated instability, evolving in the complex inner crust. The electrostatic influence arises here from all the Coulombic (charged) species (electrons + nuclei) and the self-gravitational effect from the Newtonian (gravitating) species (neutrons + nuclei). A judicious numerical analysis explores the various active accelerating/decelerating and stabilizing/destabilizing agencies of the inner crust region. It is connected that the acoustic (GNA) mode, analogously to the case of *p-*mode, plays a dominant role towards the crustal stability features before being fully collapsed up due to the dearth of nuclear fuel to counter the inward non-local self-gravity pressure effects.

In addition to the above qualitative reliability flavours, we now explore astronomical observational supports towards the investigated results. In this context, it seems noteworthy that, in the analysis of Rossi X-ray Timing Explorer (RXTE) data from the 2004 December hyperflare from SGR 1806 + 20, the global oscillation mode frequency has been found to be 625 Hz^50^. It has been interpreted that such detections link to the presence of, at least, one radial mode in the neutron star crust. This observed frequency is consistent with our analytically calculated Jeans critical frequency ($\omega_{J} = 648$ Hz). Thus, it provides a strong support and reliability to the presented GNA modal analysis. The famous space missions, CoRoT and Kepler^51^, have found mixed *p-g* modes in the red giants and revealed their deep internal structure. The *p-*mode depends on the properties on the envelope surrounding the core (outer) and the *g-*mode depends on the properties of the core structure (inner). It is believed that such space missions can detect the presence of *p-*mode, and hence, the GNA mode, in the neutron star “inner crust” region, subject to the achievement of required ultracam detection resolutions and refinements^51^. It may be conjectured that the semi-analytic model formalism presented here could enable us to identify and characterize the diversified stabilization/destabilization factors, significantly regulating the interior crustal behaviours of neutron stars and other compact astroobjects in a novel superfluidic instability perspective.

It may be noteworthy further that the instability dynamics associated with a compact spherical stellar structure, as considered herein in the GNA pattern, can undergo both the radial (central) and the non-radial (angular) oscillations relative to the reference equilibrium point. The oscillations having only the radial degrees of freedom (caused by the radial expansions and contractions) are widely termed as the stellar pulsations. It clearly means that the radial pulsations preserve the spherical geometric shape of the source object, irrespective of radial range. In other words, the original shape of the stellar structure remains free from any kind of non-radial geometric distortion factors, such as magnetic field, rotation, tidal force field, deformity effects, and so forth. It would certainly result in a pure picture of the GNA mode. Thus, it is widely seen that the spherical stellar objects remain stable and symmetric against the radial perturbations against the defined equilibrium. It is admitted herewith that a complete analysis, which is bolstered by both the polar and azimuthal coordinates describable with the help of spherical (solid) harmonics, is indeed needed in the presence of the mentioned realistic shape-complicating distortion agencies of the non-radial source^52^.

We divulge summarily that the proposed model formalism has some facts and faults, which could, indeed, be refined in our future investigations. This analysis could be appropriately extended to investigate the GNA instability, probing the interior crustal structure of neutron stars under the dynamic action of several realistic factors yet to be well considered. A number of such important active factors are the: (1) Electromagnetic forces and vortex interactions are to be properly included^8,11^; (2) Effects of the Coriolis rotations ($\Omega_{C} \sim 9.42 - 2.53 \times 10^{4}$ rad s^−1^) need inclusion^11,53^, and strong magnetic spinning ($B\sim 10^{11} - 10^{12}$ G)^11,53^; (3) Analysis is carried out here only in the low-frequency regime, the higher-order modes being auto-ignored; thereby, paving the intriguing way for inclusive refinements in the futuristic studies; (4) Relativistic dynamical effects^54^ are to be well considered; (5) Neutron superfluidic flow, both around and through the constitutive nuclei^10^, is yet to be well simulated; (6) Averaged (mean-fluidic) small-scale perturbation response characteristics are only revealed; thereby, opening an active chapter for a kinetic formalism^55^; (7) Assumed spherically symmetric geometry (gravity dominant) needs to include both the polar and azimuthal counterparts through spherical harmonics^56^; (8) Role of neutrino in the coupled modal stability is yet to be incorporated^57^; (9) Entropy wave (second sound due to superfluidity) is to be regarded well^12^; (10) Hard-core simulation platform to see the temporal evolution of the GNA instability is to be illustrated; (11) Vital possibility for a global (non-local) stability analysis with the diversified equilibrium inhomogeneities and gradient fields inclusively relevant for a fully integrated stability description is herewith opened, and so forth. At the last, we strongly believe that an appropriate comprehensive model refinement in the direction of the proposed investigation of neutron star crust characterization is needed. Inclusion of the above active factors in the model setup could elaborately depict the realistic inner crustal stability behaviours of neutron stars and similar compact  astroobjects in the complex superfluidic fabric of a unique type possibly in the years yet to come.

## Data Availability

All data generated or analysed during this study are included in this published article [and its supplementary information files].
